# Operant Self-Stimulation of Dopamine Neurons in the Substantia Nigra

**DOI:** 10.1371/journal.pone.0065799

**Published:** 2013-06-05

**Authors:** Mark A. Rossi, Tatyana Sukharnikova, Volodya Y. Hayrapetyan, Lucie Yang, Henry H. Yin

**Affiliations:** 1 Department of Psychology and Neuroscience, Duke University, Durham, North Carolina, United States of America; 2 Department of Neurobiology, Duke University, Durham, North Carolina, United States of America; 3 Center for Cognitive Neuroscience, Duke University, Durham, North Carolina, United States of America; University of Chicago, United States of America

## Abstract

We examined the contribution of the nigrostriatal DA system to instrumental learning and behavior using optogenetics in awake, behaving mice. Using Cre-inducible channelrhodopsin-2 (ChR2) in mice expressing Cre recombinase driven by the tyrosine hydroxylase promoter (Th-Cre), we tested whether selective stimulation of DA neurons in the substantia nigra pars compacta (SNC), in the absence of any natural rewards, was sufficient to promote instrumental learning in naive mice. Mice expressing ChR2 in SNC DA neurons readily learned to press a lever to receive laser stimulation, but unlike natural food rewards the lever pressing did not decline with satiation. When the number of presses required to receive a stimulation was altered, mice adjusted their rate of pressing accordingly, suggesting that the rate of stimulation was a controlled variable. Moreover, extinction, i.e. the cessation of action-contingent stimulation, and the complete reversal of the relationship between action and outcome by the imposition of an omission contingency, rapidly abolished lever pressing. Together these results suggest that selective activation of SNC DA neurons can be sufficient for acquisition and maintenance of a new instrumental action.

## Introduction

Dopamine (DA) has been implicated in motor control, learning, and motivation [1], [2], [3]. An early discovery was the capacity of DA pathway stimulation to “reinforce” operant behavior. Classic studies on intracranial self-stimulation (ICSS) showed that electrical stimulation can serve as a replacement for natural rewards like food and water to support operant conditioning. Animals can learn to perform a new action in order to stimulate various components of the DA pathway [4], [5].

The selectivity of ICSS, however, has been disputed. Electrical stimulation can activate heterogeneous cell populations as well as fibers of passage, making it difficult to stimulate any neuronal population selectively [6]. This problem has been addressed with the development of optogenetics, which made it possible to selectively stimulate dopamine neurons. Recent studies on the effects of optogenetic stimulation in the ventral tegmental area (VTA) suggest that the mesolimbic DA pathway originating in the VTA is critical for self-stimulation [7], [8], [9]. Yet it remains unknown whether the larger nigrostriatal DA pathway from the substantia nigra pars compacta (SNC) is also involved.

Here we tested whether stimulation of the nigrostriatal DA pathway supports self-stimulation with a new instrumental action. As suggested by the severe impairment in initiating voluntary actions following degeneration of SNC DA neurons in Parkinson's patients, the signal sent by these neurons is critical for voluntary behavior. Yet it is not clear whether the nigrostriatal pathway is also critical for learning new actions. In this study, we examined whether naive mice will learn to perform a new action in order to earn selective stimulation of DA neurons in the SNC. Using Cre-inducible adeno-associated viruses (AAVs) in mice expressing Cre in dopamine-synthesizing neurons, we expressed the light-activated channelrhodopsin-2 (ChR2) in SNC DA neurons. To earn stimulation of DA neurons, mice had to press a lever, an arbitrary action that is challenging to learn but well studied as an instrumental action [10].

We found that selective stimulation of SNC dopamine neurons can support learning and performance of lever pressing, in the absence of any natural rewards or motivational deprivation. We also found that lever pressing acquired with optogenetic stimulation was highly sensitive to changes in the action-outcome contingency.

## Materials and Methods

### Ethics Statement

All procedures were approved by the Institutional Animal Care and Use Committee at Duke University and followed National Institutes of Health guidelines (Protocol Number: A062-11-03).

### Mice

Naive male and female mice (*n = *20, aged 6–22 weeks at the date of surgery) were used for slice physiology and self-stimulation experiments. To express the light-gated cation channel channelrhodopsin-2 (ChR2) selectively in SNC dopaminergic neurons, we used a Cre-inducible AAV vector in mice expressing Cre recombinase under the tyrosine hydroxylase (Th) promoter (Th-Cre; B6.FVB(Cg)-Tg(Th-cre)FI172Gsat/Mmucd) [11]. Mice were injected with either Cre-inducible ChR2 (AAV5-DIO-EF1α-ChR2-YFP; *n = *8; 4 males) or Cre-inducible YFP control vector (AAV5-DIO-EF1α-YFP; *n* = 4; 3 males) [12], [13] in the SNC.

### Surgery

Viral injection and fiber implantation were performed as described previously [14]. Briefly, mice were anesthetized, and burr holes were drilled bilaterally at AP −3.2, ML ±1.6 mm relative to Bregma. A 24-gauge steel cannula was lowered at 7° relative to the dorso-ventral axis to a final depth of −4.8 mm. ChR2 or the YFP control vector (0.5 µL) were injected over 5 minutes. Injection cannulae were left in place for 5 minutes after injection to allow diffusion of the virus. Immediately after injections, custom made optic fibers (5 mm length below ferrule, 105-µm core diameter, 1.25-mm-OD ceramic zirconia ferrule; Precision Fiber Products) [15] were lowered into place ∼0.2 mm above the site of injection and secured in place with dental acrylic and skull screws. Mice were allowed to recover for 2 weeks before testing began.

### Whole-cell patch clamp recording

Th-cre mice (*n = *8 females; aged 6 weeks) were injected with ChR2 in the SNC as described above without the fiber implantation. Whole-cell recordings were performed as previously described 2–3 weeks after injection [14]. Slices were stimulated with 470-nm light from an LED assembly (Thor Labs). 10-ms flashes of light were delivered at 1–50 Hz to the entire field using a current driver (Thor Labs). Power density was estimated to be ∼5 mW/mm^2^. A MultiClamp700B amplifier (Molecular Devices) was used for all patch clamp recordings. Signals were filtered at 10-kHz and digitized at 20-kHz with a Digidata 1440A digitizer (Molecular Devices).

### Operant behavior

**Figure 1:**
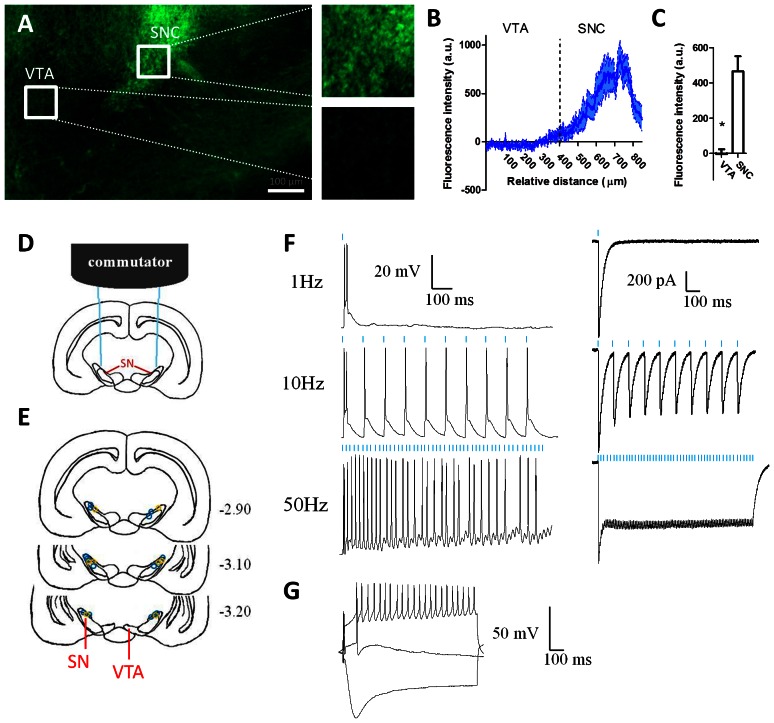
ChR2 expression in substantia nigra dopamine neurons. (A) ChR2 was limited to the SNC with no expression in the neighboring VTA. Fluorescence intensity (with background subtracted) was calculated along a line through the VTA and SNC. Scale bar is 100 µm. (B) Average (± s.e.m.; 8 mice) normalized fluorescence values along the extent of the line from VTA to SNC. (C) Average intensity values from VTA and SNC (*n* = 8, *p*<0.001). (D) Schematic representation showing two implanted optic fibers targeting the SN connected to a rotating commutator. (E) The location of injection sites is shown for ChR2 mice (blue circles; *n* = 8) and YFP controls (yellow circles; *n* = 4). The numbers indicate the anterior-posterior location (mm from Bregma). (F) *In vitro* stimulation of DA neurons. Current-clamp recordings (left column) from a ChR2-expressing dopamine neuron in the substantia nigra: 10-ms pulses of 470-nm light (blue lines) elicit frequency-dependent spiking at 1-Hz, 10-Hz, and 50-Hz. In voltage-clamp recordings (right column), light pulses induce inward currents in ChR2-expressing dopamine neurons. (G) Current injection experiments showed a sag in response to hyperpolarizing current indicating a hyperpolarization activated current (I_h_), characteristic of SNC DA neurons.

All tests took place in standard operant chambers (Med Associates) as previously described [16]. Before testing each day, mice were connected to a 473-nm wavelength laser by two sheathed fibers (62-µm core diameter; 21-inch length; connected by ceramic sleeves, Precision Fiber Products). The fibers extended from the implants on the mouse through the top of the operant chamber to a rotating optical commutator (Doric) that split a single laser beam into two beams for bilateral stimulation (**Fig. 1D**). The total output of the laser was adjusted each day, to obtain ∼20 mW transmittance into the brain. All behavioral tests lasted 60 minutes, during which one lever was ‘active’ and one was ‘inactive’, unless otherwise noted. For discrete trial FR1 sessions, pressing either lever resulted in retraction of both levers for 5-s, but pressing the active lever resulted in a 5-s, 50-Hz pulse train (10-ms square pulses). Pressing the inactive lever yielded no stimulation.

Mice were trained for at least 10 daily sessions. Once lever pressing was acquired, “satiety” tests were conducted, which consisted of two consecutive FR1 sessions separated by ∼5 minutes (*n* = 8; 5-s, 50-Hz pulse train). The effect of stimulation frequency (1, 10, or 50-Hz; 10-ms square pulse width, 5-s pulse train) on lever pressing was examined in three sessions per mouse (*n* = 8).

During fixed ratio 3 (FR3) and fixed ratio 5 (FR5) sessions (*n = *4), pressing the active lever 3 or 5 times, respectively, resulted in retraction of the levers and initiation of laser stimulation (5-s, 50-Hz). Mice were tested for three sessions each at FR1, FR3, and FR5 and then returned to FR1 for one session. For the progressive ratio 5 (PR5) test, the number of presses required to earn one stimulation increased by five each time a stimulation was earned. The session ended after 30 minutes.

During the 60-min extinction session (*n* = 6), pressing either lever resulted in retraction of both levers for 5-s, but no stimulation was delivered. Omission testing (n = 4) took place over three daily sessions during which time laser stimulation (5-s pulse train, 50-Hz, 10-ms square pulse) was delivered once every 10-s if the mice did not press the lever. Each time the lever was pressed, the 15-s timeout was reset, so the only way for mice to receive stimulation was to refrain from pressing the formerly active lever.

Duration differentiation training consisted of six daily 60-min sessions. Mice (*n = *4) were able to press and hold the lever for as long as they wanted. As long as the lever was depressed, the laser pulsed at 50-Hz (10-ms pulses). Behavioral experiments are described in the order they were performed.

### Histology

Mice were anesthetized with isoflurane and perfused with ice-cold 4% paraformaldehyde. Brains were sliced at 60-µm and examined using fluorescent microscopy (Axio Zoom.v16, Zeiss) to confirm the expression of ChR2. Using the Zeiss Zen software, fluorescence intensity within the SNC and VTA was calculated by drawing a line extending through the VTA and SNC. The background intensity values were subtracted from the pixel intensity values.

## Results

TH-cre mice were injected with ChR2 or YFP bilaterally targeting the substantia nigra. Histological analysis revealed that expression was limited to the pars compacta and pars reticulata regions (**Fig. 1A–E**). Fluorescence intensity was measured along a line extending from the VTA through the SNC (**Fig. 1B**). There was no evidence of viral expression in the VTA (**Fig. 1C**; paired *t*-test, *p<*0.001).

### In vitro stimulation of dopamine neurons

Using whole-cell patch clamp recordings from visually-identified ChR2-expressing neurons in acute brain slices, we verified that laser stimulation of SNC DA neurons expressing Cre-inducible ChR2 was sufficient to produce reliable spiking in these neurons (**Fig. 1F**). In current clamp mode, current injection experiments showed a sag in response to hyperpolarizing current indicating a hyperpolarization activated current (I_h_), a slow-developing inward current characteristic of SNC DA neurons (**Fig. 1G**) [17], [18]. DA neurons spiked reliably in response to 10-ms pulses of 470-nm light at 1-Hz, 10-Hz, and 50-Hz. Likewise, using voltage clamp recording, light pulses produced reliable frequency-dependent inward currents [9].

### Stimulation of nigrostriatal dopamine release is sufficient to promote operant conditioning

**Figure 2:**
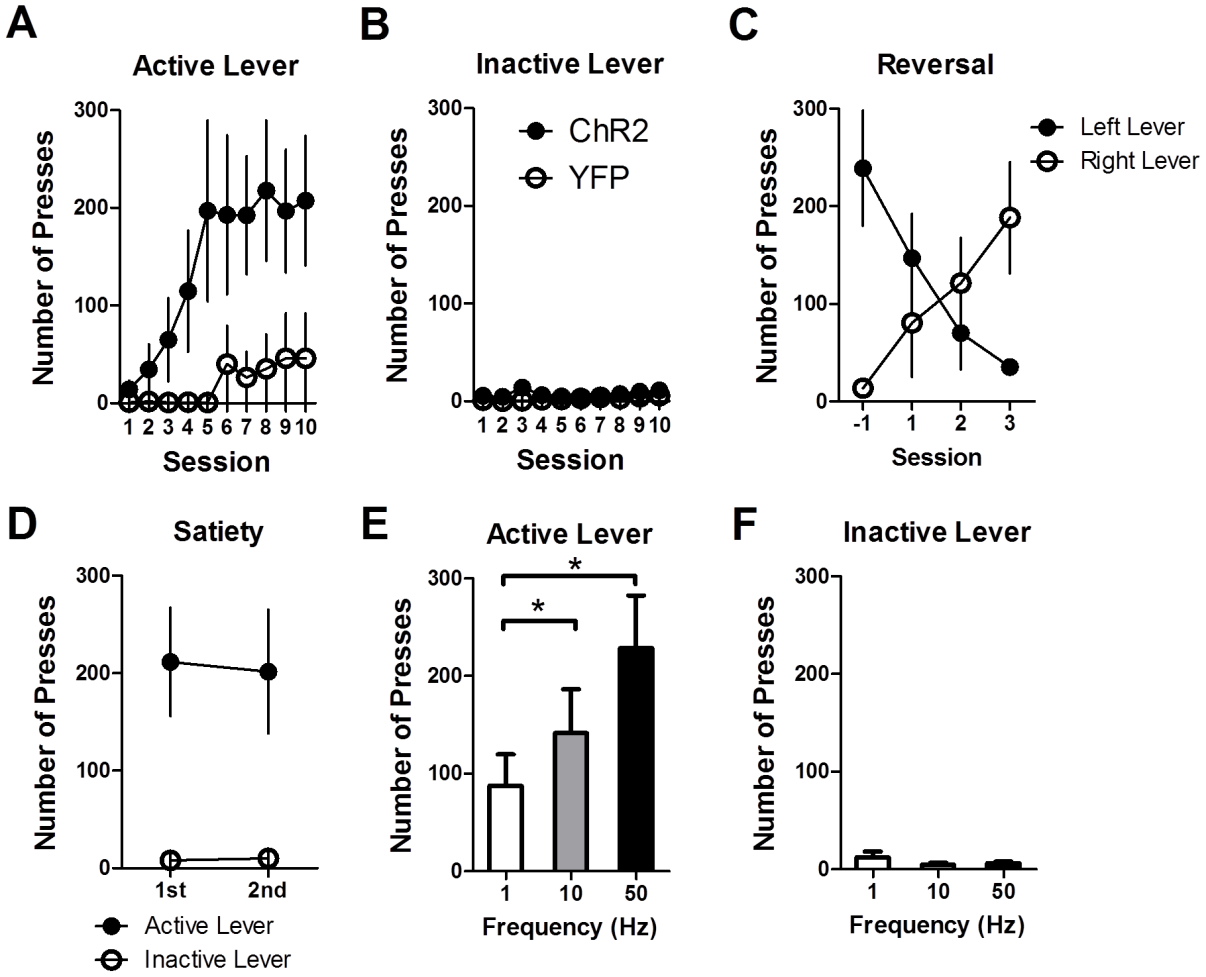
Operant self-stimulation of nigral dopamine neurons. The number of active (A) and inactive (B) lever presses during the first 10 testing sessions: ChR2-expressing mice rapidly acquire lever pressing for dopamine stimulation, whereas YFP control mice do not. (C) Following reversal of the active and inactive levers, mice (*n = *3) changed their preference. (D) There was no effect of “satiety.” Self-stimulation behavior did not decrease when the animals received a second session immediately after the first. (E) Mice press the active lever less frequently for 1-Hz stimulation than they do for 50-Hz and 10-Hz stimulation (* p<0.05). (F) The number of inactive presses is not affected by stimulation frequency. Values are mean ± s.e.m.

To test whether stimulation of DA neurons immediately following an action was sufficient to produce operant conditioning, we placed mice in operant chambers in which pressing one lever (‘active’ lever) resulted in bilateral laser stimulation (5-s, 50-Hz), whereas pressing the other lever (‘inactive’ lever) yielded no stimulation. Any lever press resulted in retraction of both levers for a 5-s timeout period. Mice expressing ChR2 in DA neurons rapidly increased the rate of active lever presses, whereas those expressing YFP only did not. ChR2 mice pressed the active lever significantly more than YFP control mice (**Fig. 2A**; two-way ANOVA, Group [ChR2 or YFP]×Session: no main effect of session, *F*_(9,93)_<1.0, *p*>0.05; main effect of Group, *F*_(1,93)_ = 14.70, *p*<0.001; no interaction, *F*_(9,93)_<1.0, *p*>0.05). All ChR2 mice preferred the active lever (**Fig. 2B**).

When the active and inactive levers were switched, mice readily adjusted their behavior and began pressing the newly active lever (**Fig. 2C**; two-way RM-ANOVA, Lever [Left or Right]×Session: no main effects, *F*<1.0, *p*>0.05; interaction between Lever and Session, *F*_(3,12)_ = 11.14, *p<*0.001). Activation of SNC DA neurons is indeed sufficient to generate robust operant self-stimulation behavior.

We next tested if self-stimulation behavior was sensitive to the effects of “satiety” (**Fig. 2D**). ChR2-expressing mice were allowed to self-stimulate for two consecutive 60-minute sessions. Neither the number of active nor inactive presses was reduced (**Fig. 2D**; paired *t*-test, *t*_(7)_>1.0, *p*>0.05), i.e. press rate remained the same despite repeated stimulation, suggesting that operant self-stimulation is not significantly reduced by “satiety.”

We then tested whether self-stimulation was sensitive to the frequency of laser stimulation. We found that the number of active lever presses varied as a function of frequency (**Fig. 2E**; one-way RM-ANOVA, *F*_(2,14)_ = 12.24, *p*<0.001). *Post hoc* test confirmed that the number of active presses for 1-Hz stimulation was lower compared to 10-Hz and 50-Hz stimulation (*p*<0.05). Stimulation frequency did not affect the number of inactive presses (**Fig. 2F**; one-way RM-ANOVA, *F*_(2,14)_ = 1.46, *p*>0.05).

**Figure 3:**
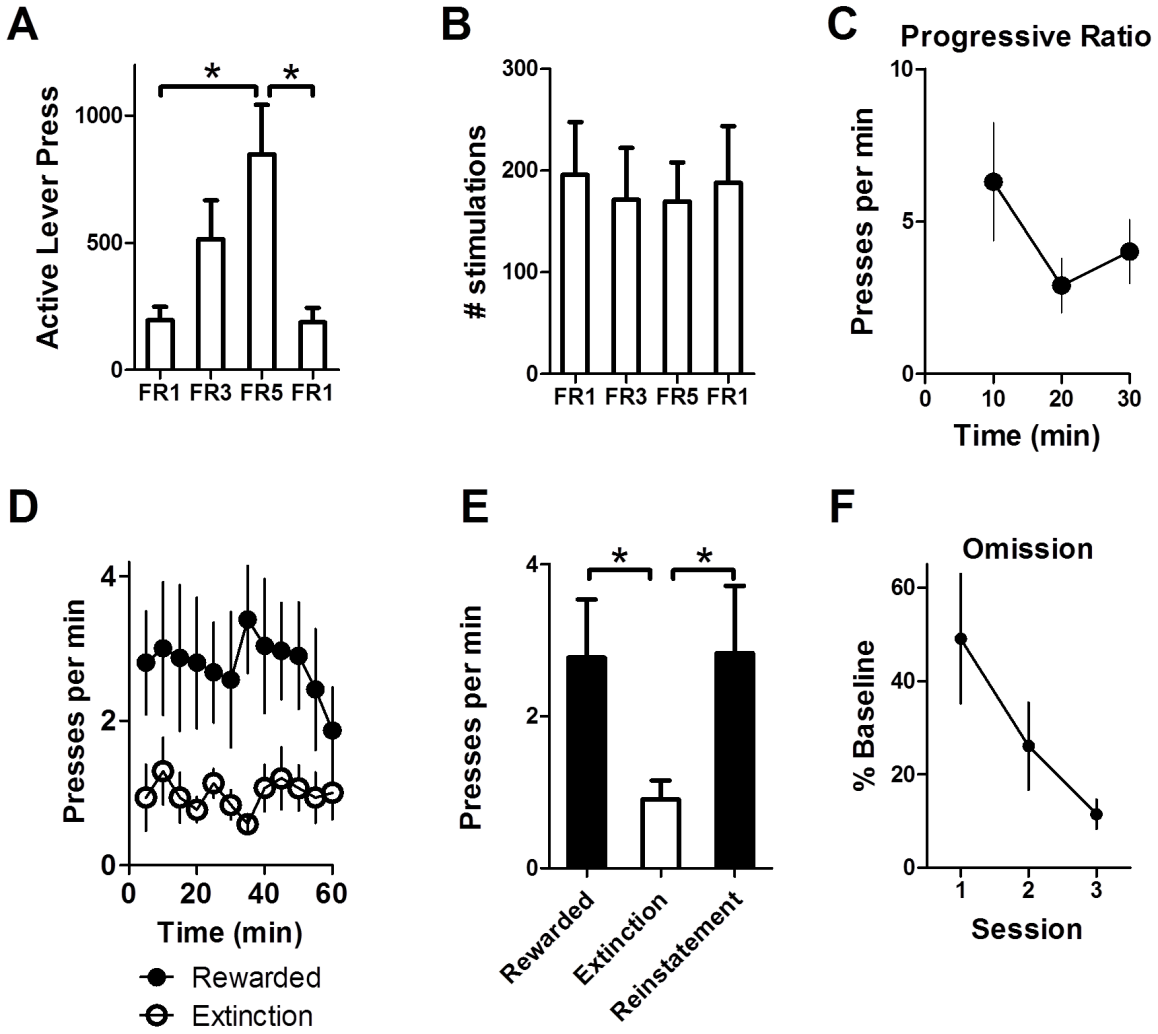
Self-stimulation of nigra DA neurons is sensitive to changes in contingency. (A) Mice (*n* = 4) increase their rate of lever pressing when the ratio requirement (number of presses needed to earn one stimulation) is changed. (B) The number of stimulations received remains unchanged when the ratio requirement is changed. (C) On a progressive ratio 5 task (PR5), ChR2 mice (*n* = 6) reduce pressing for stimulation. (D) Lever pressing is rapidly extinguished when stimulation is no longer available (*n* = 6). (E) Rate of pressing during extinction is reduced compared to pre-extinction rewarded sessions. The rate of pressing returns to baseline level when the contingency between lever pressing and laser stimulation is restored. (F) Omission (*n* = 4) reduces the number of active lever presses (FR1 press rate is used as baseline).

To understand the role of the instrumental contingency between action and outcome in self-stimulation, we manipulated the schedule of reinforcement. Mice acquired operant self-stimulation on fixed ratio 1 (FR1; one press results in one stimulation). When the schedule of reinforcement was changed from FR1 to FR3 and then FR5 (3 or 5 presses result in 1 stimulation, respectively), mice increased their rate of pressing accordingly (**Fig. 3A**). The steady-state number of active lever presses is shown for FR1, FR3, FR5. Press rate returned to FR1 levels once the schedule of reinforcement was returned to FR1 (one-way RM-ANOVA, *F*_(3,9)_ = 12.27, *p*<0.01). The number of stimulations received was unchanged when the schedule of reinforcement was changed (**Fig. 3B**; *F*_(3,9)_<1.0, *p*>0.05).

We then used a progressive ratio 5 (PR5) schedule, in which the number of presses required to earn one stimulation increased by five following each stimulation. ChR2-expressing mice quickly reduced the rate of pressing (**Fig. 3C**; one-way RM ANOVA, *F*_(2,10)_ = 4.53, *p*<0.05).

To assess the sensitivity of dopamine self-stimulation to more radical changes in the action-outcome contingency, we conducted an extinction session in which pressing the lever resulted in lever retraction, as during the training session, but no laser stimulation. Under extinction, the rate of pressing was immediately reduced, unlike a more gradual decline in lever pressing for natural foods (**Fig. 3D**). **Fig. 3E** shows the average rate of pressing under rewarded, extinction, and reinstatement conditions (one-way RM-ANOVA: *F*_(2,10)_ = 8.55, *p*<0.01). Tukey's Multiple Comparison tests showed that the rate of pressing during extinction was significantly lower than that during rewarded sessions (*p*<0.05), but returned to pre-extinction levels during a reinstatement session, when stimulation once again followed lever pressing (*p>*0.05).

We next imposed an omission contingency in which DA stimulation was automatically delivered every 10 seconds if the mice refrained from pressing the active lever, but pressing the active lever delayed the stimulation by 15 seconds. When the instrumental contingency was thus reversed, all mice quickly reduced the number of presses during three consecutive omission sessions (**Fig. 3F**; RM-ANOVA: *F*_(2,6)_ = 5.94, *p*<0.05).

**Figure 4:**
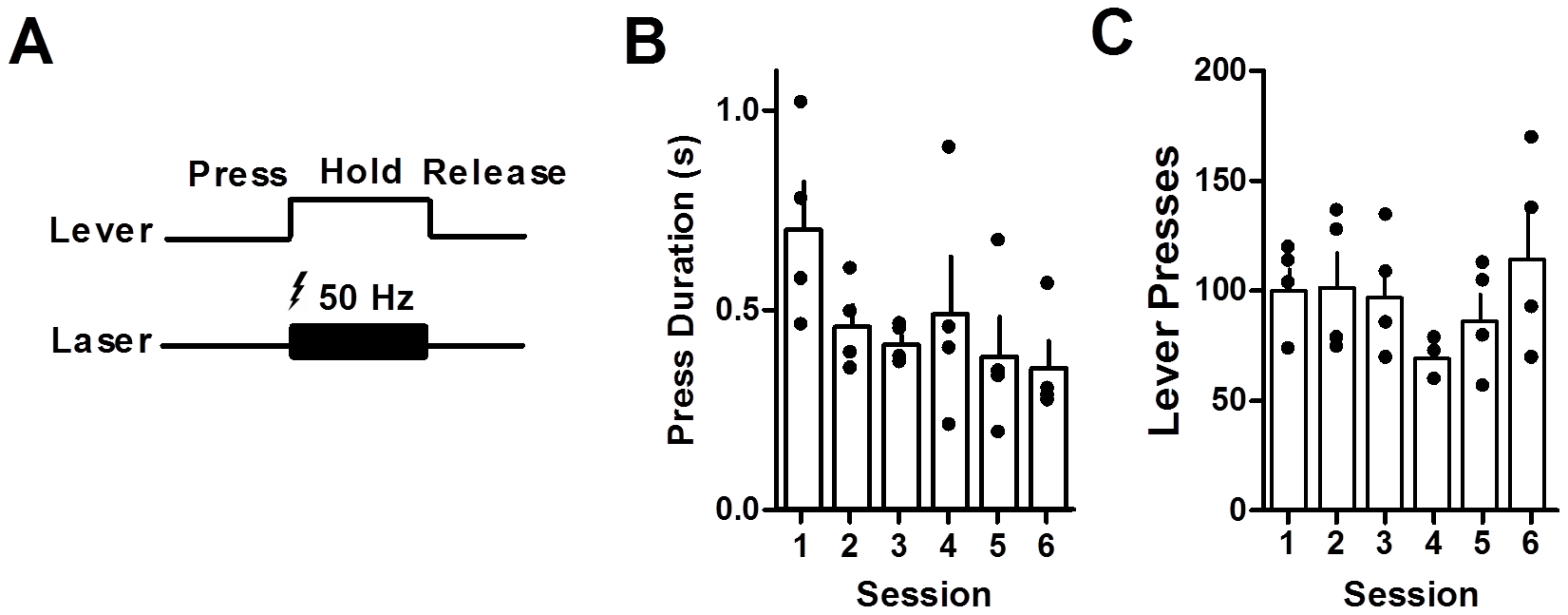
Duration differentiation. (A) The laser is turned on (50 Hz) as long as the mice (*n* = 4) hold down the lever. (B) Press duration decreases with training, demonstrating that mice are unable to learn to hold the lever down in order to receive extended stimulation. (C) Total number of presses is unaffected by training. For panels B and C, individual data (filled circles) are superimposed over mean+s.e.m. values.

To test whether animals would learn to sustain the immediate sensation of optogenetic stimulation, mice were tested on a duration differentiation task: the laser provided 50-Hz stimulation as long as the lever was held down (**Fig. 4A**). If the mice perceived DA stimulation as immediately pleasurable, then with training they would increase the duration of the lever press. However, this was not observed. In fact, mice actually decreased the duration of their presses over time (**Fig. 4B**; one-way RM-ANOVA, *F*_(3,15)_ = 3.74, *p<*0.05), while the total number of presses remained unchanged (**Fig. 4C**; *F*_(5,15)_ = 1.14, *p*>0.05).

## Discussion

Our results demonstrate that selective stimulation of DA neurons in the SNC is sufficient to support the acquisition and expression of lever pressing behavior in naive mice. As shown in **Fig. 2C**, when the active and inactive levers were reversed, the behavior of the animal also readily reversed, demonstrating high sensitivity to the action-outcome contingency.

When the instrumental contingency was altered by changing the ratio requirement (i.e. number of presses required per reward), the rate of lever pressing was also adjusted accordingly (**Fig. 3A**). The rate of stimulation, however, remained stable (**Fig. 3B**), suggesting that it may be a regulated variable, i.e. mice can vary behavioral output in order to obtain a desired overall rate of stimulation. With the cessation of stimulation, we observed rapid extinction (**Fig. 3D–E**), which was also seen in classic work on ICSS [19]. In addition, when the instrumental contingency was reversed by the imposition of an omission contingency, the mice also reduced their lever pressing (**Fig. 3F**), suggesting that the observed behavior is under the control of the action-outcome contingency. Interestingly, the reduction in lever pressing under extinction was much more rapid than that observed under omission or lever reversal contingencies. This pattern is not seen in traditional operant conditioning with natural food rewards, which typically shows more gradual extinction, but frequently observed in ICSS. When the stimulation does not follow lever pressing, DA signaling may still promote lever pressing to a certain extent. But such motivational arousal is not found when stimulation is omitted altogether, as in extinction. There is still no convincing explanation for this difference.

It is possible that pressing the lever simply produced an immediate sensation of pleasure–one of the original explanations for self-stimulation behavior. Yet we were able to rule out this possibility using the duration differentiation procedure in which the animal has control over the duration of stimulation [20], [21]. Stimulation of SNC DA neurons did not create an immediate “reward” that the animal wants to prolong by holding down the lever (**Fig. 4**). When the stimulation was continued for as long as the animal chose to hold down the lever, the duration of lever pressing actually decreased. These results suggest that dopamine stimulation does not simply correspond to a reward signal, though we cannot rule out the possibility that a change in the dopamine signal, such as a sharp decline, is the pleasure signal that animals attempt to obtain by pressing.

Despite the lack of immediate pleasure, naive mice with no food deprivation could learn a new action, though the duration differentiation experiment did not rule out the possibility that stimulation of SNC DA neurons produced a delayed sense of pleasure that reinforced the operant behavior. Regardless of the actual mechanisms, the ChR2 stimulation allowed completely naive mice to discover the appropriate action that led to the stimulation. Once acquired, lever pressing could also be maintained indefinitely with stimulation, in the absence of any other motivational source. These results suggest that DA neurons in the SNC play an important role in initial instrumental learning.

Our results agree with recent work showing self-stimulation of VTA DA in rats [9]. Witten et al. also manipulated the instrumental contingency and found that nose poking reinforced by stimulation of DA neurons in the VTA was reduced by contingency degradation. On the other hand, another recent study showed that optogenetic stimulation of VTA DA neurons in mice could only reactivate previously extinguished nose poking behavior, but by itself is not sufficient to produce operant conditioning, in the absence of food rewards [8]. The extent of ChR2 expression or differences in stimulation parameters may account for the discrepancies [9], [22].

One clear difference between our study and recent studies on the VTA is in the behavioral measure, as the other studies all used nose poke. Compared to lever pressing, nose poking in rodents is much easier to acquire, as it is already in the animal's natural repertoire and more susceptible to the influences of Pavlovian stimulus-outcome contingencies [10]. It has been suggested that the mesolimbic DA system contributes mainly to instrumental performance, in particular to the effort animals are willing to spend for a given food reward [23]. That is, stimulation of VTA DA neurons may boost performance of the instrumental action, but is not critical for the initial learning of that action. On the other hand, stimulation of SNC DA neurons, which project heavily to dorsal striatal regions critical for learning and performance of instrumental actions, can produce the type of plasticity required for initial instrumental learning. More systematic examination of the location of stimulation and the efficacy of behavior is needed to shed light on the differences, if any, between VTA and SNC stimulation, and the respective roles these DA neuronal populations play in learning and behavior.

It should be noted that the optical stimulation used in our lever press acquisition experiments cannot be equated with the activation of DA neurons under natural conditions. Although DA neurons can fire rapidly, without a direct comparison of DA release caused by optic stimulation and natural DA release during operant conditioning, we cannot conclude that the DA signal in self-stimulation experiments is comparable to what happens under natural conditions. Moreover, the stimulation of SNC DA neurons should not be equated with DA release *per se*. In addition to DA, other transmitters such as glutamate and GABA have been shown to be co-released from Th-positive neurons [24], [25]. Stimulation of SNC DA neurons may provide additional, non-dopaminergic, signals to other brain regions that are critical for instrumental learning [26]. Additional work on the effects of optogenetic stimulation on neural circuits receiving dopaminergic projections is therefore needed to elucidate the downstream changes accompanying instrumental learning and performance.
